# First complete mitochondrial genome of the *Corydoras pygmaeus* (Actinopteri: Callichthyidae) and its phylogenetic implications

**DOI:** 10.1080/23802359.2022.2122750

**Published:** 2022-09-15

**Authors:** Huajun Zhang, Li-An Gao, Wenlei Zhang

**Affiliations:** School of Tourism and Resource Environment, Qiannan Normal University for Nationalities, Duyun, China

**Keywords:** Next-generation sequencing, mitogenome, mouse fish, phylogeny, ornamental fish

## Abstract

The Pygmy corydoras *Corydoras pygmaeus* Knaack, 1966, is the smallest member of the genus *Corydoras*, belonging to the family Callichthyidae and order Siluriformes. The complete mitochondrial genome of *C. pygmaeus* was sequenced and assembled using next-generation sequencing technology, and phylogenetically compared with those of other species of this genus. The mitochondrial genome of *C. pygmaeus* is a circular DNA molecule with a size of 16,840 bp (GenBank no. ON729306). A phylogenetic tree was constructed based on 13 protein-coding genes of *C. pygmaeus* and 13 species of the family Callichthyidae, which showed that *C. pygmaeus* clustered with other species of this genus, but was the first branch to differentiate. These results could provide basic data for phylogenetic analysis and population genetic diversity protection of *Corydoras* and Callichthyidae fish in the future.

## Introduction

**Figure 1.**
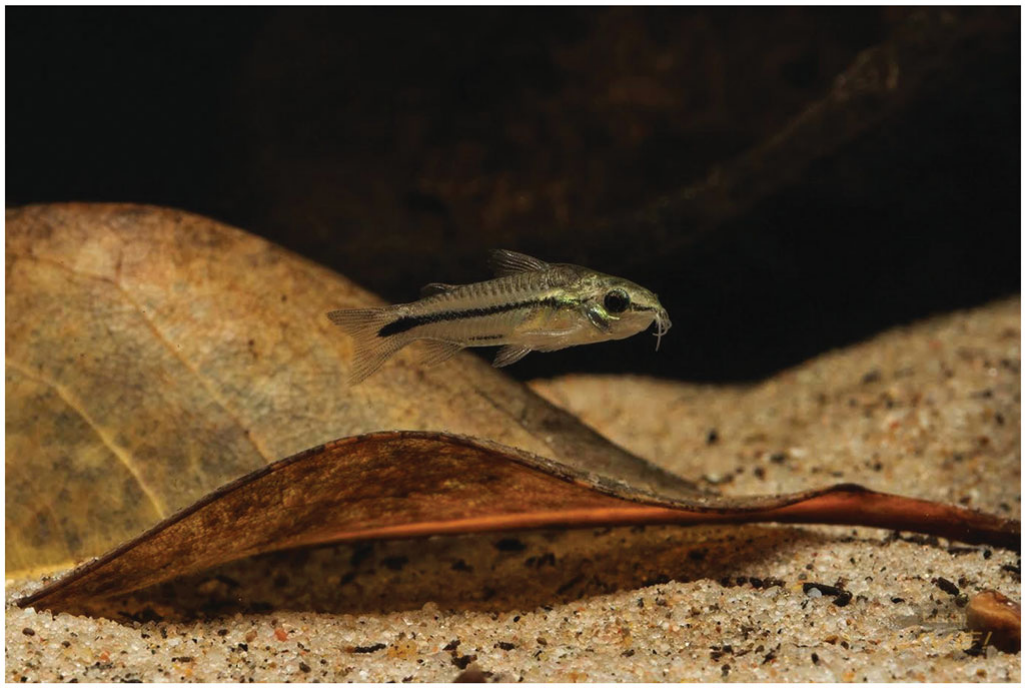
Photograph of *Corydoras pygmaeus.*

Compared to nuclear genes, mitochondrial DNA (mtDNA) is maternally inherited, undergoes rapid evolution, and small in size, making it an important molecular marker for research on fish evolutionary genetics, molecular ecology, species identification, and conservation biology (Sun et al. 2020). Mouse fish are members of the genera *Aspidoras*, *Brochis*, and *Corydoras* of the subfamily Corydoradinae. They have two cute ‘moustaches’ next to their mouths and look like small mice swimming in water, and thus the name ‘mouse fish.’ Mouse fish are typical benthic and migratory fish with omnivorous feeding habits. They are found in South America. Almost all the main and tributary water systems of the Amazon River contain pikes, but the composition of species in the watersheds where various species are distributed is different (Huysentruyt and Adriaens 2005; Liu et al. 2019; Lv et al. 2020; Tencatt et al. 2021; Sun et al. 2022). For instance, most mouse fish gather in the middle and lower reaches, where the water flow is relatively gentle, and a few strong swift warriors live in the upper reaches of the river. The Pygmy corydoras *Corydoras pygmaeus* (Figure 1) Knaack, 1966, is the smallest member of the genus *Corydoras.*

## Materials

The sample was collected from Duyun City, Guizhou, China (26°17′31.15″ N, 107°31′13.02″ E) on June 10, 2021. This study was reviewed and approved by the Animal Ethics Committee of the Qiannan Normal University for Nationalities. Voucher specimens were stored at the School of Tourism and Resource Environment, Qiannan Normal University for Nationalities, Guizhou, China, under the voucher number C_pygmaeus_A (https://www.sgmtu.edu.cn/, Huajun Zhang, 155046257@qq.com).

## Methods

DNA extraction was performed using a column animal genome DNA extraction kit according to the manufacturer’s protocol (Sun et al. 2021). Sequencing of the mitochondrial genome was performed on an Illumina Novaseq 6000 platform and assembled using GetOrganelle 1.7.5 (Jin et al. 2020). Genes in the sequence were annotated using MitoAnnotator (Iwasaki et al. 2013) (http://mitofish.aori.u-tokyo.ac.jp/annotation/input.html) and MITOS (http://mitos.bioinf.uni-leipzig.de/index.py). The tRNAscan-SE 1.21 (Lowe and Eddy 1997) (http://lowelab.ucsc.edu/tRNAscan-SE/index.html) online tool was used to predict the secondary structure of tRNA, which was corrected manually and finally submitted to GenBank (Accession no. ON729306). To determine the phylogenetic significance of *C. pygmaeus* in the genus *Corydoras*, 11 other species of this genus were used to reconstruct the phylogenetic tree based on 13 protein-coding genes, using *Hemigrammus erythrozonus* as the outgroup (Sun et al. 2021). The phylogenetic tree was constructed using the maximum likelihood method with IQ-TREE v1.6.8 under the edge-linked partition model for 5,000 standard bootstraps.

## Results

**Figure 2.**
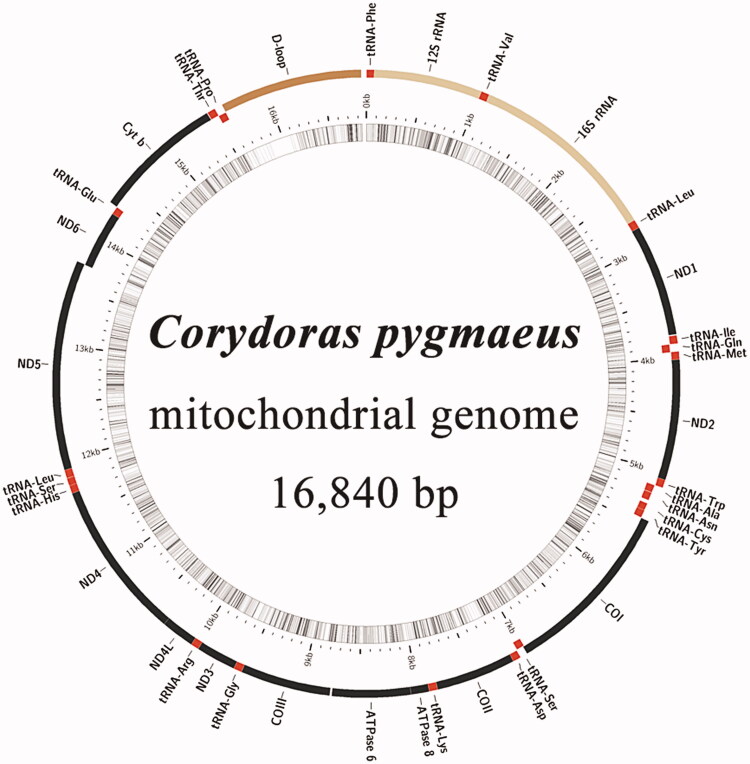
Gene map of the *Corydoras pygmaeus* mitochondrial genome.

The complete mitochondrial genome of *C. pygmaeus* is 16,840 bp in length (Figure 2), with a nucleotide composition of 33.25% A, 25.37% C, 14.39% G, and 26.98% T, and a higher A + T (60.24%) than G + C (39.76%) content. The mitochondrial genome is composed of a control region, 2 rRNA genes, 13 protein-coding genes, and 22 tRNA genes. Among these 37 genes, 9 are encoded by the light strand, while 28 are encoded by the heavy strand. All 13 protein-coding genes start with the codon ATG, except for *COI*, which starts with the codon GTG. Typical stop codons (TAG and TAA) were observed for most protein-coding genes, except for incomplete terminal codons T for *ND2*, *COII*, *COIII*, *ND3*, and *ND4*; AGA for *ND5*; and AGG for *COI*.

**Figure 3.**
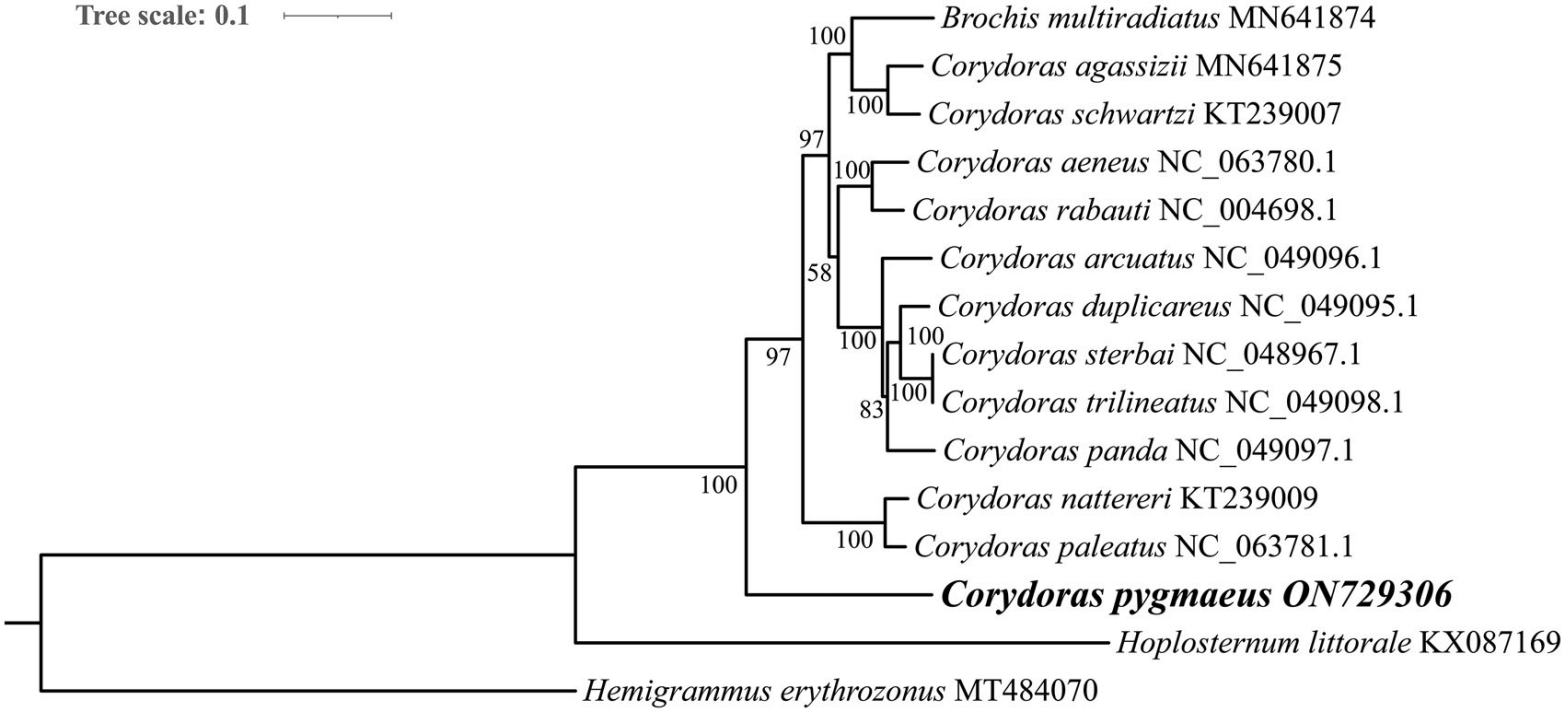
Maximum likelihood tree of 14 Callichthyidae species and one outgroup based on 13 protein-coding genes. The accession numbers are listed after the species names. The *C. pygmaeus* mitochondrial genome is marked in bold font.

Phylogenetic analysis showed that Callichthyidae species formed a branch and were well separated from the outgroup, and that *Brochis multiradiatus* clustered with other species of the genus *Corydoras* before *C. pygmaeus* (Figure 3). The target species *C. pygmaeus* clustered with other species of this genus, but was the first branch to differentiate.

## Discussion and conclusion

In this study, the composition and structure of *C. pygmaeus* mtDNA were predicted and analyzed to provide basic data for phylogenetic analysis and population genetic diversity protection of *Corydoras* and Callichthyidae fish in the future.

## Data Availability

The data that support the findings of this study are openly available in GenBank of NCBI at (https://www.ncbi.nlm.nih.gov/) under the accession no. ON729306. The associated BioProject, SRA, and Bio-Sample numbers are PRJNA847725, SRR19612627, and SAMN28952161, respectively.
